# Supplementary material for: Clinical Characteristics and Outcomes of Persistent Staphylococcal Bacteremia in a Tertiary Care Hospital
Source: Antibiotics (Basel). 2023 Feb 24;12(3):454. doi: 10.3390/antibiotics12030454 (PMC10044455; doi:10.3390/antibiotics12030454)
Supplement: Supplementary file 1 [file antibiotics-12-00454-s001.zip › antibiotics-2215761-supplementary.pdf]

antibiotics

Article

Clinical Characteristics and Outcomes of Persistent Staphylococcal Bacteremia in a Tertiary Care Hospital

Shiori Kitaya [1,2,*,†], Hajime Kanamori [1,*,†], Yukio Katori [2] and Koichi Tokuda [1]

1 Department of Infectious Diseases, Internal Medicine, Tohoku University Graduate School of Medicine, Sendai 980-8574, Japan
2 Department of Otolaryngology, Head and Neck Surgery, Tohoku University Graduate School of Medicine, Sendai 980-8574, Japan
* Correspondence: shiori.kitaya.b7@tohoku.ac.jp (S.K.); kanamori@med.tohoku.ac.jp (H.K.); Tel.: +81-22-717-7373 (S.K.)
† These authors contributed equally to this work.

Table S1. Clinical characteristics of persistent *Staphylococcus aureus* bacteremia, persistent *Staphylococcus epidermidis* bacteremia, and other persistent coagulase negative staphylococci bacteremia.

	Persistent *S. aureus* bacteremia (*n* = 92)	Persistent *S. epidermidis* bacteremia (*n* = 62)	Persistent CoNS bacteremia (*n* = 26)	*p*– Value	Corrected *p*-Value		
					Persistent *S. aureus* group vs. Persistent *S. epidermidis* group	Persistent *S. aureus* group vs. Persistent CoNS group	Persistent *S. epidermidis* group vs. Persistent CoNS group
**Demography**							
Sex (male, %)	65 (70.7)	32 (51.6)	16 (61.5)				
Age, years, median (IQR)	63.5 (56.5–69.3)	44.5 (34.0–54.5)	58.5 (58.3–58.8)	0.003		0.004	
**Comorbidities**							
Diabetes mellitus	25 (27.2)	7 (11.3)	2 (7.7)	0.017			
ESDR on hemodialysis	11 (12.0)	4 (6.5)	2 (7.7)				
Liver cirrhosis	13 (14.1)	5 (8.1)	2 (7.7)				
Solid malignancy	28 (30.4)	17 (27.4)	7 (26.9)				
Hematologic malignancy	2 (2.2)	11 (17.7)	4 (15.4)	0.001	0.003	0.042	
Neutropenia	1 (1.1)	4 (6.5)	3 (11.5)	0.036			
Immunosuppression	12 (13.0)	18 (29.0)	6 (23.1)	0.043			
**Vital signs**							
BMI, kg/m², median (IQR)	21.1 (18.4–23.8)	22.2 (17.5–24.3)	22.0 (18.9–23.4)				
Body temperature, °C, median (IQR)	38.7 (38.0–39.1) (*n* = 82)	38.0 (37.5–38.6) (*n* = 58)	38.7 (37.6–39.3) (*n* = 84)	0.007	0.005		

**Laboratory markers**						
White blood cell count, 10^9/L, median (IQR)	9550.0 (7375.0–12,200.0)	8000.0 (4325.0–12,400.0)	7850.0 (4550.0–12,800.0)			
Neutrophil count, 10^9/L, median (IQR)	8160.0 (6210.0–10,950.0) (*n* = 89)	6030.0 (3725.0–10,950.0) (*n* = 56)	5380.0 (3070.0–9730.0) (*n* = 25)	0.019		
C-reactive protein, mg/dL, median (IQR)	10.0 (4.6–16.9)	5.3 (1.3–9.1)	3.0 (1.7–5.4)	<0.001	<0.001	<0.001
**Devices**						
Intravascular device	53 (57.6)	55 (88.7)	21 (80.8)	<0.001	<0.001	
Intravascular device removal	46 (86.8)	43 (78.2)	15 (71.4)			
Cardiovascular surgery	28 (30.4)	10 (16.1)	4 (15.4)			
ECMO	0 (0)	1 (1.6)	1 (3.8)			
Continuous hemodiafiltration	0 (0)	15 (24.2)	4 (15.4)			
Mechanical ventilation	24 (26.1)	18 (29.0)	2 (7.7)			
**Status of persistent bacteremia**						
Period until FUBC is carried out, median (IQR)	3.0 (2.0–4.0)	3.0 (1.0–4.8)	3.0 (1.0–4.8)			
Duration of bacteremia, median (IQR)	3.5 (2.0–6.3)	3.0 (1.3–7.0)	4.0 (1.3–7.0)			
**Site of infection**						
CRBSI	40 (29.2)	55 (82.1)	18 (66.7)	<0.001	<0.001	<0.001
Abscess	15 (10.9)	1 (1.5)	0 (0)	0.018		

Infectious endocarditis	14 (10.2)	1 (1.5)	1 (3.7)	0.048	
Endovascular devices infections	12 (8.8)	0 (0)	1 (3.7)	0.024	0.029
Septic embolism	10 (7.3)	1 (1.5)	0 (0)		
Pyogenic spondylitis	10 (7.3)	1 (1.5)	0 (0)		
Thrombophlebitis	9 (6.6)	3 (4.5)	1 (3.7)		
Surgical site infection	5 (3.6)	0 (0)	0 (0)		
Suppurative arthritis	4 (2.9)	0 (0)	0 (0)		
Skin and soft tissue infections	3 (2.2)	1 (1.5)	0 (0)		
Osteomyelitis	2 (1.5)	0 (0)	1 (3.7)		
Others	4 (2.9)	0 (0)	0 (0)		
Unknown	9 (6.6)	4 (6.0)	5 (18.5)		
**Hospital stay**					
Duration of hospitalization, days, median (IQR)	59.5 (36.8–104.3)	80.5 (39.5–128.0)	65.0 (40.0–104.0)		
Presence of ICU	28 (30.4)	30 (48.4)	10 (38.5)		
Duration of ICU stay, days, median (IQR)	0 (0–8)	0 (0–23.0)	0 (0–18.3)		
Presence of HCU	6 (6.5)	4 (6.5)	3 (11.5)		
Duration of HCU stay, days, median (IQR)	0 (0–0)	0 (0–0)	0 (0–0)		
Presence of CCU	6 (6.5)	0 (0)	1 (3.8)		
Duration of CCU stay, days, median (IQR)	0 (0–0)	0 (0–0)	0 (0–0)		

**Intervention**			
Use of antibiotics (Appropriate)	76 (82.6)	57 (91.9)	24 (92.3)
Source control	61 (66.3)	50 (80.6)	16 (61.5)
**Mortality**			
Early (30-day) mortality	5 (5.4)	2 (3.2)	0 (0)
Late (30–90 days) mortality	2 (2.2)	4 (6.5)	0 (0)
90-day mortality	7 (7.6)	6 (9.7)	0 (0)

Data are presented as number (%), unless indicated otherwise. *P*-values are listed in the table only for those that showed significant differences. The blood test was performed on the same day of blood culture collection. Immunosuppression was considered in the presence of neutropenia, hematopoietic stem-cell transplantation, solid organ transplantation, and corticosteroid therapy (prednisone 16 mg per day for 15 days). Cardiovascular surgery includes valve replacement, vascular graft replacement, ventricular assist device, and cardiac device implantation. Endovascular device infections encompass those of vascular grafts, pacemakers, implantable cardioverter defibrillators, and left ventricular assist devices. Persistent CoNS bacteremia includes persistent bacteremia caused by *Staphylococcus hominis* (*n* = 8), *Staphylococcus lugdunensis* (*n* = 6), *Staphylococcus capitis* (*n* = 4), *Staphylococcus caprae* (*n* = 3), *Staphylococcus warneri* (*n* = 2), *Staphylococcus haemolyticus* (*n* = 2), and *Staphylococcus piscifermentans* (*n* = 1). BMI, body mass index; CCU, coronary care unit; CI, confidence intervals; CoNS, coagulase negative staphylococci; CRBSI, catheter-related blood stream infection; ECMO, extracorporeal membrane oxygenation; ESDR, end-stage renal disease; FUBC, follow-up blood culture; HCU, high care unit; ICU, intensive care unit; IQR, interquartile range; *S. aureus*, *Staphylococcus aureus*; *S. epidermidis*, *Staphylococcus epidermidis*.
